# Can insecticide-treated netting provide protection for Equids from *Culicoides* biting midges in the United Kingdom?

**DOI:** 10.1186/s13071-015-1182-x

**Published:** 2015-11-25

**Authors:** Tiffany Baker, Simon Carpenter, Simon Gubbins, Richard Newton, Giovanni Lo Iacono, James Wood, Lara Ellen Harrup

**Affiliations:** University of Surrey, Guildford, Surrey, GU2 7XH UK; Vector-borne Viral Diseases Programme, The Pirbright Institute, Ash Road, Pirbright, Surrey, GU24 0NF UK; Animal Health Trust, Lanwades Park, Kentford, Newmarket, Suffolk, CB8 7UU UK; Department of Veterinary Medicine, University of Cambridge, Madingley Road, Cambridge, CB3 0ES UK

**Keywords:** *Culicoides*, WHO cone bioassay, African horse sickness virus, Seasonal recurrent allergic dermatitis, Sweet itch

## Abstract

**Background:**

Biting midges of the genus *Culicoides* Latreille, 1809 (Diptera: Ceratopogonidae) cause a significant biting nuisance to equines and are responsible for the biological transmission of African horse sickness virus (AHSV). While currently restricted in distribution to sub-Saharan Africa, AHSV has a history of emergence into southern Europe and causes one of the most lethal diseases of horses and other species of Equidae. In the event of an outbreak of AHSV, the use of insecticide treated nets (ITNs) to screen equine accomodation is recommended by competent authorities including the Office International des Épizooties (OIE) in order to reduce vector-host contact.

**Methods:**

Seven commercially avaliable pyrethroid insecticides and three repellent compounds, all of which are licensed for amateur use, were assessed in modified World Health Organization (WHO) cone bioassay trials in the laboratory using a colony line of *Culicoides nubeculosus* (Meigen), 1830. Two field trials were subsequently conducted to test the efficiency of treated net screens in preventing entry of *Culicoides*.

**Results:**

A formulation of cypermethrin (0.15 % *w/w*) and pyrethrins (0.2 % *w/w*) (Tri-Tec 14®, LS Sales (Farnham) Ltd, Bloxham, UK) applied to black polyvinyl-coated polyester insect screen (1.6 mm aperture; 1.6 mm thickness) inflicted 100 % mortality on batches of *C. nubeculosus* following a three minute exposure in the WHO cone bioassays at 1, 7 and 14 days post-treatment. Tri-Tec 14® outperformed all other treatments tested and was subsequently selected for use in field trials. The first trial demonstrated that treated screens placed around an ultraviolet light-suction trap entirely prevented *Culicoides* being collected, despite their collection in identical traps with untreated screening or no screening. The second field trial examined entry of *Culicoides* into stables containing horses and found that while the insecticide treated screens reduced entry substantially, there was still a small risk of exposure to biting.

**Conclusions:**

Screened stables can be utilised as part of an integrated control program in the event of an AHSV outbreak in order to reduce vector-host contact and may also be applicable to protection of horses from *Culicoides* during transport.

## Background

Biting midges of the genus *Culicoides* Latreille, 1809 (Diptera: Ceratopogonidae) cause biting nuisance to humans, livestock and equines [1]. Hypersensitivity reaction to the bites of *Culicoides* are the primary cause of equine summer seasonal recurrent allergic dermatitis [2], commonly known as ‘sweet-itch’. This condition affects between 2 and 12 % of horses in the UK [2–5], although studies from other countries in northern Europe indicates that this prevalence may be underestimated [6]. *Culicoides* also act as the biological vectors of arboviruses, including African horse sickness virus (AHSV), which effects all Equidae and causes devastating outbreaks of the disease African horse sickness (AHS) (reviewed by [7, 8]). Mortality rates in horses, mules and European and Asian donkeys can reach 95, 50 and 10 % respectively in susceptible populations dependent upon the form of the clinical manifestation of the disease (in ascending order of severity: the subclinical form (horse sickness fever), the subacute/cardiac form, the mixed (cardiac and pulmonary form) and the acute respiratory/pulmonary form), while zebra and African donkeys rarely exhibit clinical signs [7]. While currently restricted to sub-Saharan Africa, AHSV has long been recognised as a threat to the equine industry in Europe due to historical incursions of the virus in 1966 (Spain) and in 1987–1991 (Spain and Portugal) [9, 10]. Recent outbreaks of bluetongue virus (BTV), a related *Culicoides*-borne arbovirus of ruminants has heightened this awareness and led to dedicated risk assessments, contingency planning and legislation for the incursion and spread of AHSV [11–13]. A large-scale epidemic of AHSV in the UK could potentially cost the equine industry up to £3.5 billion and the traumatic nature of the disease would also be expected to have a severe social impact [14].

While vaccines for AHSV are available [15], none are currently licenced for use within the UK or European Union (EU), where AHSV is currently absent but is compulsorily notifiable. Although available vaccines may be granted licences for emergency use in the event of an AHSV incursion into Europe, in their absence, a combination of effective vector control measures and animal movement restrictions is the only currently available means of reducing AHSV spread following incursion [16]. While these measures were notably unsuccessful in controlling BTV outbreaks in northern Europe during the epidemic of BTV-8 (2006–9) [17], the fewer available hosts for AHSV in the region may improve the potential for techniques to reduce vector-host contact rates and mitigate against virus transmission.

Among the methods used to control *Culicoides* populations worldwide (reviewed by [7, 16, 18]), housing of horses during periods of peak biting activity has long been anecdotally observed to act as a protective measure against AHS [19]. The success of this method in reducing ASHV transmission in the Republic of South Africa (RSA) is attributed to the strong exophilic nature of the principle AHSV vector in the region, *C. imicola* Kieffer, 1913 [20]. In more high-lying regions of RSA, however, the presence of the endophilic species *C. bolitinos* Meiswinkel, 1989 sustains AHSV transmission even when horses are stabled, although the number of *Culicoides* collected were substantially reduced by screening [20].

In northern Europe, members of the subgenus *Avaritia* (which in the UK includes: *C. obsoletus* (Meigen), 1818; *C. scoticus* Downes and Kettle*,* 1952; *C. dewulfi* (Goetghebuer), 1936; and *C. chiopterus* (Meigen), 1830) are the most likely vectors of BTV [21, 22] and the recently identified Schmallenberg virus (SBV) [23]. In addition, isolations of AHSV were also made from pools of species including *C. obsoletus* and/or *C. scoticus* in Cadiz, Spain during the epidemic in 1988 [24]. While primarily exophilic, these species are thought to exhibit seasonably variable levels of endophilic behaviour [25–27]. This indicates that additional measures may be required to protect stabled horses from vector contact [28, 29].

The creation of truly vector-proof accommodation is rarely attempted due to the costs involved and the difficulties in accurately monitoring potential lapses in biosecurity. The creation or modification of equine accommodation to reduce vector-host contact, however, may be achievable if wide-scale stockholder uptake is required in the event of an AHSV outbreak. It is currently recommended by competent authorities that in the event of an AHSV incursion, stables and horse transport should be screened with netting treated with an insecticide with a residual effect [30, 31]. There are, however, no insecticidal products currently authorised specifically against *Culicoides* in the EU [32]. In addition, no quantitative data is available regarding the effect of available insecticides in reducing vector-host contact, or on the logistical feasibility of their use.

A current key concern is that changes in health and safety and environmental legislation for the use of insecticides in the UK and the EU have resulted in insecticides previously recommended for use against *Culicoides* no longer being licenced [18]. In addition, other insecticides are now only available for use by those holding a current Certificate of Competence in the use of pesticides [33, 34], something the majority of UK horse owners are unlikely to hold. This study therefore aims to investigate the effectiveness of mesh netting suitable for screening stables treated with commercially available pyrethroid insecticides licenced by the UK Health and Safety Executive (HSE) for ‘amateur use’ for reducing vector-host contact.

## Methods

### Insecticides and screening

**Fig. 1:**
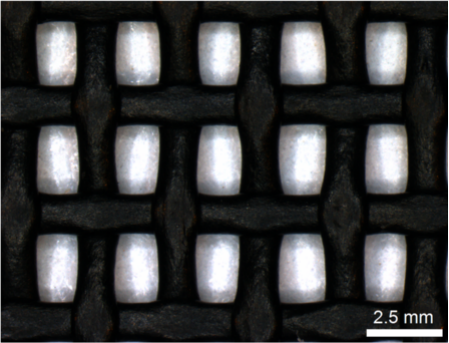
Black polyvinyl-coated polyester used during studies. Image taken and calibrated using a QICAM Fast 1394 digital camera (QImaging, Surrey, BC, Canada) and Image-Pro Insight (MediaCybernetics, Rockville, MD, USA) mounted on a Leica M80 stereo light microscope (Leica Microsystems, Milton Keynes, UK)

The UK HSE Control of Pesticides Regulation database [35] and manufactures’ information was screened to select insecticides which matched the following criteria: (*i*) contains at least one pyrethroid as an active ingredient, (*ii*) are licenced for ‘amateur use’, (*iii*) are currently commercially available in the UK, (*iv*) are marketed as being capable of being used to treat surfaces/buildings for the reduction of flying insects and (*v*) in order to be financially viable for the treatment of large surface areas available in quantities of 2 L or greater. This resulted in the selection of seven insecticidal treatments (Table 1). In addition, three repellents representative of the compounds commonly used in the UK equine market (Harrup et al., unpublished data) were selected for comparison (Table 1). PetMesh insect screen (Fine Mesh Metals, Telford, UK) black polyvinyl-coated polyester (1.6 mm aperture; 1.6 mm thickness) (Fig. 1) was used for all experiments.

**Table 1:** World Health Organisation (WHO) Cone Bioassays: Treatments compared using modified WHO Cone Bioassays

Treatment	Type	Treatment name (Supplier)	Active ingredient
A	Insecticide	Agropharm’s Dairy Fly Spray (Agropharm Ltd, Penn, UK)	Pyrethrins including cinerins 0.25 % *w/w* ^a^
B	Insecticide	Degrain Insectaclear C (Lodi UK, Kingswinform, UK)	Cypermethrin 0.1 % *w/w*
C	Insecticide	Fly Free Zone (Fly Away Ltd, Stourbridge, UK)	Permethrin 0.1 % *w/w*; Tetramethrin 0.04 % *w/w*
D	Insecticide	Protector C (Agropharm Ltd, Penn, UK)	Cypermethrin 0.09 % *w/w*
E	Insecticide	Strikeback Insect Killing Spray (Group 55, Preston, UK)	Cypermethrin 0.01 % *w/w*
F	Insecticide	Tri-Tec 14® (LS Sales (Farnham) Ltd, Bloxham, UK)	Cypermethrin 0.15 % *w/w*; Pyrethrins 0.2 % *w/w* ^a^
G	Insecticide	Ultrashield EX (W.F. Young, Inc, East Longmeadow, MA, USA)	Permethrin 0.5 % *w/w*; Pyrethrins 0.1 % *w/w* ^a^
H	Repellent	NAF Off Citronella (Greencoat Ltd t/a Natural Animal Feeds, Monmouth, UK)	Citronella Oil <1.5 % *w/w*
I	Repellent	NAF Off DEET POWER (Greencoat Ltd t/a Natural Animal Feeds, Monmouth, UK)	DEET <20 % *w/w*
J	Repellent	NAF Off Extra Effect (Greencoat Ltd t/a Natural Animal Feeds,Monmouth, UK)	Citriodiol1% *w/w*
K	-	Untreated Mesh	
L	-	Untreated Filter Paper	

^a^also contains Piperonylbutoxide [5-[2-(2-butoxyethoxy)ethoxymethyl]-6-propyl-1-3-benzodioxole as a synergist

### WHO cone bioassays

Initial screening of insecticide efficacy was conducted using colony derived *Culicoides nubeculosus* (Meigen), 1830 from the Pirbright line [36] and modified World Health Organization (WHO) cone bioassays (Fig. 1). Sections of mesh netting (10 cm × 30 cm) were individually treated with each of the selected insecticides/repellents on day 0 (one mesh section per insecticide/repellent). Each mesh section was sprayed for 10s (5 s per side) with the selected insecticide/repellent dispensed from a 1.5 L hand-held pump sprayers (Pest Control Shop, Preston, UK). In addition, each mesh section was weighed pre- and post-treatment using a portable balance (SPU123: Ohaus Corporation, NJ, USA) and the weight of insecticide applied calculated in order to estimate the amount of active ingredient applied (Table 2). Following treatment all treated mesh sections in addition to an untreated mesh section were hung undercover outdoors for 24 h prior to any exposure assays being conducted to allow the treatments to dry. Mesh sections were hung at least 30 cm apart to prevent cross-contamination and protected from direct sunlight and rainfall, but the cover was open on all four sides allowing mesh sections to be exposed to the prevailing weather conditions. Treatment of mesh sections was conducted by LEH, followed by blinding and randomisation by SC, all subsequent exposure and feeding experiments were conducted by LEH.

**Table 2:** World Health Organisation (WHO) Cone Bioassays: Mean estimated application rate of active ingredients in mg/cm^2^ of selected insecticide/repellent-based treatments, range between treatment batches shown in parenthesis

Treatment	Active Ingredient (mg/cm^2^)						
	Citriodiol	Citronella Oil	Cypermethrin	DEET	Permethrin	Pyrethrins	Tetramethrin
A^a^	-	-	-	-	-	2.8 (2.2–3.3)	-
B	-	-	1.2 (1.1–1.4)	-	-	-	-
C	-	-	-	-	1.2 (1.1–1.4)	-	0.5 (0.4–0.6)
D	-	-	1.0 (0.9–1.1)	-	-	-	-
E	-	-	0.1 (0.1–0.1)	-	-	-	-
F^a^	-	-	2.7 (2.0–3.0)	-	-	3.1 (2.3–3.4)	-
G^a^	-	-	-	-	6.8 (6.6–7.2)	1.3 (1.3–1.4)	-
H	-	22.1 (19.0–27.5)	-	-	-	-	-
I	-	-	-	292.2 (215.1–366.6)	-	-	-
J	12.5 (10.6–14.7)	-	-	-	-	-	-

− not an active ingredient in treatment

^a^also contains Piperonylbutoxide [5-[2-(2-butoxyethoxy)ethoxymethyl]-6-propyl-1-3-benzodioxole as a synergist) (Treatments K and L untreated negative controls

**Fig. 2:**
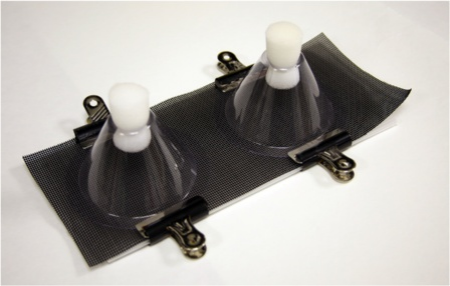
World Health Organization (WHO) cone bioassays exposure equipment. Treated or untreated mesh net sections secured between 10 cm by 30 cm white translucent plastic board (Challoner Marketing Ltd, Amersham, UK) and two WHO bioassay cones (Vector Control Research Unit, UniversitiSains Malaysia, Malaysia) into which twenty-five three to four day-old non-bloodfed female *C. nubeculosus* were introduced per bioassay cone for a three minute exposure period

At 24 h (day 1), seven days (day 7) and 14 days (day 14) post-treatment mesh sections were secured between 10 cm by 30 cm white translucent plastic boards (Challoner Marketing Ltd, Amersham, UK) and two WHO bioassay cones (Vector Control Research Unit, UniversitiSains Malaysia, Malaysia) were prepared for each mesh section (Fig. 2). Twenty-five three to four day-old non-bloodfed female *C. nubeculosus*, which had had access to cotton wool soaked in a 10 % sucrose solution for the previous 24 h were introduced to each bioassay cone. The entrance port to each WHO bioassay cone was then sealed with a 25 mm polyurethane white foam stopper (Fisher Scientific, UK) (Fig. 2). After a three minute exposure period, *Culicoides* were removed from the bioassay cone using a manual aspirator fitted with an in-line HEPA-filter (GE Healthcare Life Sciences, UK) and transferred to an 8 cm round cardboard pill pot (Watkins and Doncaster, UK) covered with fine white nylon mesh (160 μm aperture) (MegaView Science Co. Ltd, Taiwan). The above protocol was also repeated in duplicate using untreated white filter paper (Whatman’s No. 1: GE Healthcare Life Sciences, UK) as an additional negative control (the randomised treated mesh sections already contain an untreated mesh as a primary negative control).

The resulting 24 pots containing ‘exposed’ *Culicoides* were then incubated at 27 °C (+/− 2 °C) and 70 %rH (+/−5 %) for 24 h in an incubator (model 303NP: LMS Ltd, UK) with additional humidity supplied via a Vicks Mini Ultra Humidifier (model: VH5000E1; Proctor & Gamble, UK). In order to allow blood feeding responses to be tested post-incubation, the exposed *Culicoides* were not given access to sucrose solution during the incubation period. Following the 24 h incubation, pots containing the ‘exposed’ *Culicoides* were removed from the incubator and the number of live (capable of oriented movement) and dead (motionless) *Culicoides* in each pot were recorded. To record sub-lethal anti-feedant effects of exposure, each pot containing at least one live *Culicoides* was given access for one hour to defibrinated horse blood (TCS Biosciences, UK) supplied and warmed to 37 °C via a Hemotek Membrane Feeding System (Hemotek, UK) with stretched Parafilm M® membrane (Bemis Company Inc., WI, USA). Following the one hour feeding period all *Culicoides* were killed by prolonged exposure to cold (−20 °C), the contents of each pot were then examined under a stereomicroscope (x4-40 magnification) and the number of fed and unfed *Culicoides* recorded. The temperature (°C) and relative humidity (% rH) during the 24 h incubation and the one hour feeding period were monitored and recorded using TinyTag View 2 data loggers (Gemini Data Loggers Ltd, UK).

The above treatment protocol and cone bioassay protocol was repeated in triplicate to account for variation in colony *Culicoides* batch, weather conditions or treatment application. This resulted in a total of three mesh sections/filter paper controls for each treatment or control (hereafter referred to as the ‘treatment batches’) with two bioassay cones for each mesh section or filter paper control (within-mesh section replicates), with 25 *C. nubeculosus* per bioassay cone, resulting in a total of 150 *C. nubeculosus* being exposed in each treatment or control.

#### Statistical analysis

Generalised Linear Mixed Models (GLMM) with a Binomial error distribution and a logit link function were implemented in a Bayesian setting using the bglmer function in package ‘blme’ version 1.0-2 [37] in R v 3.1.2 [38] in order to investigate the effect of treatment on *C. nubeculosus* mortality and feeding rate. The GLMMs were fitted by maximum likelihood with the Laplace approximation with flat covariance priors and normal fixed priors, with *product* and *days since treatment* considered as fixed effects and *treatment batch* and *within-mesh section replicate* as nested random effects to take into account any variation between batches and replicates not accounted for by the fixed effects. In models of *C. nubeculosus* feeding rate, *mortality rate* was also included as a fixed effect to account for variation in the number of surviving *Culicoides* present able to potentially feed. Final models were obtained using a backwards-stepwise-selection-based procedure [39], such that variables that did not contribute significantly to explaining variation in mortality rate or feeding rate were successively eliminated on the basis of Akaike Information Criterion (AIC) [40]. This continued until the removal of a variable caused an increase in AIC of two or more. Differences in mortality rates and feeding rate between treatments were then assessed using multiple Tukey’s all-pair comparisons using the ‘glht’ function in package multcomp version 1.3-7 [41].

### Field trials

A treatment was selected on the basis of causing the highest mortality rate and/or greatest reduction in feeding rate during the modified WHO bioassays. In order to assess the efficacy of this treatment under field conditions, two sets of experiments were conducted at a polo club in Hampshire, UK (51.2414° N, −1.6645° W) between June and August 2014. This location was selected with prior knowledge of a large number of horses being present exhibiting clinical signs of summer seasonal recurrent allergic dermatitis. Weather conditions (air temperature (°C), relative humidity (%), rainfall (mm), wind speed (ms^−1^), wind direction (°), and solar radiation (wm^-2^)) during all field trials were recorded every 15 min, using an automatic weather station (Decagon Devices, Pullman, WA, USA), and summarized as mean values across each overnight trapping period. Wind direction is a circular variable, with the constraint that directions of 0°and 360° represent the same direction. Hence the mean transformed wind direction for each trapping period was calculated using the mean angle trigonometric such that *transformed wind direction* = *ATAN*2(*sin*(*wind direction*), *cos*(*wind direction*)) ∗ (180/*π*)).

#### Field trial one

**Fig. 3:**
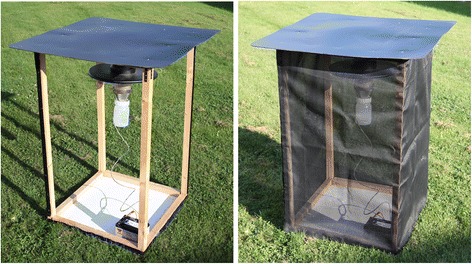
Field Trial One: Equipment utilised to investigate the effectiveness of insecticide-treated mesh in preventing entry of *Culicoides*. Ultraviolet (UV) Center for Disease Control (CDC) light-suction trap (model 912: John W Hock, FL, USA) hung within an open-sided wooded frame (width: 1.0 m; length: 1.0 m; height: 1.2 m), with solid black roofs extending 10 cm in each direction, and solid white wooden base. The sides of each frame were either: (*i*) covered with insecticide-treated mesh, (*ii*) covered with untreated mesh, or (*iii*) left uncovered without mesh

The first field trial utilised a randomised Latin square design to assess the effectiveness of insecticide-treated nets to prevent *Culicoides* entry under field conditions over an extended time period. Ultraviolet (UV) Center for Disease Control (CDC) light-suction traps (model 912: John W Hock, FL, USA) were hung within three open-sided wooded frames (width: 1.0 m; length: 1.0 m; height: 1.2 m), with solid black roofs extending 10 cm in each direction, and solid white wooden bases (one trap per frame) (Fig. 3). The sides of each frame were either: (*i*) covered with insecticide-treated mesh, (*ii*) covered with untreated mesh, or (*iii*) left uncovered without mesh (Fig. 3). Mesh panels were secured to the wooden frame using 2 cm wide stick-on Velcro® (RS Components, Corby, UK), in addition to the adhesive glue on the Velcro®, each strip was sewed onto the mesh panels with black cotton thread to increase adhesion. Mesh panels were treated using a 1.5 L hand-held pump sprayer (Pest Control Shop, Preston, UK) on day 0 and attached to the wooden frames. Untreated mesh panels were also attached to the wooden frames on day 0. Overnight collections using the UV CDC traps in each of the three frames were then made on day 1, 3, 5, 7, 9, 11, 13 and 15. Insects were collected into water with a drop of non-bleaching detergent (Hederol: Proctor and Gamble Professional, UK), then transferred to 70 % ethanol for storage prior to identification. Any *Culicoides* which had passed through the mesh, but had not been collected in the light-trap were aspirated from the base of the wooden frame using a battery powered aspirator (Watkins and Doncaster, UK) and stored in 70 % ethanol prior to identification. The above procedure was repeated in triplicate resulting in 24 overnight collections for each treatment (eight per treatment batch) and 72 collections in total. Frames were rotated between trap locations with their treatments to prevent any potential cross-contamination, and placed at least 50 m apart.

#### Field trial two

**Fig. 4:**
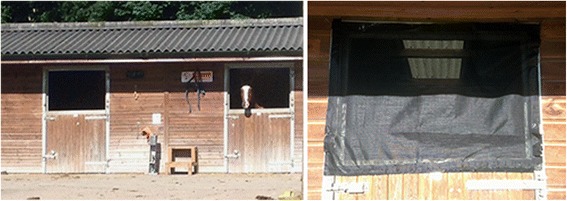
Field Trial Two: Stable type utilised in field trial two to investigate the effectiveness of insecticide-treated mesh in preventing entry of *Culicoides* as measured by miniature Ultraviolet (UV) Center for Disease Control (CDC) light-suction traps. One UV light-suction trap was located within each stable whose entrance was either (*i*) covered with insecticide-treated mesh, (*ii*) covered with untreated mesh, or (*iii*) left uncovered without mesh

The second field trial utilised a three by three randomised Latin square design. Ultraviolet CDC light-suction traps (model 912: John W Hock, Gainsville, FL, USA) were hung within three stables on the stable yard, one trap per stable. The entrance to each stable was then either (*i*) covered with insecticide-treated mesh, (*ii*) covered with untreated mesh, or (*iii*) left uncovered without mesh (Fig. 4). Mesh panels were secured to the stable doors using 5 cm wide heavy-duty stick-on Velcro®(Velcro Ltd, Middlewich, UK) and each strip was sewed onto the mesh panels with black cotton thread to increase adhesion. Mesh panels were treated using a 1.5 L hand-held pump sprayer (Pest Control Shop, Preston, UK) on day 0 and attached to the stable doors. Untreated mesh panels were also attached to the stable doors on day 0. Overnight collections using the UV CDC traps in each of the three stables were then made on day 1, 2 and 3. Insects were collected into water with a drop of non-bleaching detergent (Hederol: Proctor and Gamble Professional, UK) then transferred to 70 % ethanol for storage prior to identification. Nine overnight collections were carried out for each treatment and 27 in total. Each stable contained one horse and doors were not opened while the UV light-traps were operational, with the horses present within the stables for the duration of the overnight collection periods. Horses were not rotated with treatments ensuring each treatment was tested with each horse/stable combination three times in a cross-over design allowing any inter-horse variation in their attractiveness to *Culicoides* to be accounted for in the statistical analysis*.*

### Culicoides *species identification*

*Culicoides* were separated from other arthropods using a stereo microscope (10-40X) and then further identified to species-level based on wing morphology [42]. Female specimens of the subgenus *Avaritia* species were identified to group level morphologically and then identified to species level using a multiplex polymerase chain reaction (PCR) assay [43]. Total DNA was extracted from individual *Culicoides* specimens using a non-destructive DNA extraction technique [44]. *Culicoides* were individually incubated in 200 μl of DXT Tissue Digest Reagent (Qiagen, Crawley, UK) with 1 % Proteinase K (Qiagen, Crawley, UK) for 16 h at 40 °C. *Culicoides* were then transferred individually from the tissue digest solution to 70 % ethanol and stored at 4 °C for future analysis. The remaining tissue digest solution was then incubated at 70 °C for 15 min to inactivate the proteinase K and then ethanol precipitated to remove PCR inhibitors using either Pellet Paint® Co-Precipitant (Merck Millipore, Darmstadt, Germany) or glycogen from *Mytilus edulis* (Roche, UK) as a co-precipitant to improve DNA yield. The purified DNA extractions were resuspended in 100 μl of 10 mM Tris HCL pH 8.0 (Buffer EB: Qiagen, Crawley, UK).

PCR amplification was conducted in a GeneAmp 9700 thermal cycler (Applied Biosystems, UK). Reactions were performed in a total of 12 μl consisting of 0.4 μl nuclease free water, 6.0 μl TopTaq mastermix (Qiagen, UK), 1.2 μl CoralLoad concentrate (Qiagen, UK), 1.2 μl D- (+)-Trehalose, 0.2 μl 20 mM *C. obsoletus* specific forward primers, (obsF 5′ TGCAGGAGCTTCTGTAGATTTG 3′) [45], 0.4 μl 20 mM *C. scoticus* specific forward primer (ScoF 5′ ACCGGCATAACTTTTGATCG 3′) [45], 0.2 μl 20 mM *C. chiopterus* specific forward primer (ChiF 5′ TACCGCCCTCTTATCACCCTA 3′) [45], 0.2 μl 20 mM *C. dewulfi* specific primer (DewF 5′ ATACTAGGAGCGCCCGACAT 3′) [45], and 1.0 μl 20 mM *Culicoides* universal reverse primer COIR (5′ CAGGTAAAATTAAAATATAAACTTCTGG 3′) [43] and 2 μl DNA template. Positive and negative controls for the amplification reactions were carried out at every PCR round. The PCR cycling conditions were as follows: an initial denaturation step at 94 °C for 3 min followed by 35 cycles of 94 °C for 30 s, 58 °C for 30 s, 72 °C for 1 min, followed by a final extension step at 72 °C for 10 min. Amplification was assessed by electrophoresis of PCR products on 2 % (*w/v*) pre-cast agrose gels containing SYBR Safe (E-Gel® 96: Life Technologies, UK) run for seven minutes. Gels were visualised and imaged using Chemi-Doc MP system (BioRad, UK). E-gel® images were then edited using the E-editor system (Life Technologies, UK), and banding patterns compared to each other along with positive controls, and by comparison with E-Gel® Low Range Quantitative DNA Ladder (100–2000 bp:Life Technologies, UK) to allow species composition based on the following expected band sizes: *C. obsoletus* 355 bp; *C. scoticus*: 229 bp; *C. dewulfi*: 493 bp; *C. chiopterus*: 435 bp.

#### Statistical analysis

**Fig. 5:**
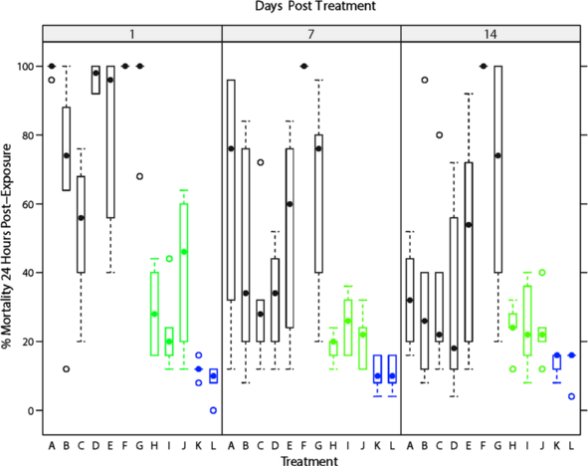
World Health Organisation (WHO) Cone Bioassays: Box-and-whisker plots of percentage mortality in *C. nubeculosus* 24 h post-exposure and split by treatment and days since treatment. Variation within the box-and-whisker plots represents variance in mortality rates between treatment batches and between within-mesh section replicates. Median values shown as filled black circles, outliers as hollow black circles, the interquartile range as hollow rectangles (black: insecticide-based treatments; green: repellent-based treatments; blue: negative controls)

Generalised Linear Mixed Models (GLMM) with a Binomial error distribution and a logit link function were implemented in a Bayesian setting using the bglmer function in package ‘blme’ version 1.0-2 [37] in R v 3.1.2 [38] in order to investigate the effect of treatment on the number of *Culicoides* and specifically the number of females of potential AHSV vector species of *Culicoides* (*C. obsoletus*; *C. scoticus*; *C. dewulfi*; *C. chiopterus*) collected within UV CDC light-suction traps inside frames (field trial one) or inside stables (field trial two) which are either: (*i*) covered with insecticide-treated mesh, (*ii*) covered with untreated mesh, or (*iii*) left uncovered, i.e. no mesh (Fig. 5). The GLMMs were fitted by maximum likelihood with the Laplace approximation with flat covariance priors and normal fixed priors, with *treatment batch* included as a random effect treatment, *trap location*, *days post-treatment* and meteorological conditions comprising a total of five predictors considered as additional fixed predicators. Meteorological predictors were: *mean air temperature* (°C), *mean humidity* (% rH), *precipitation* (mm), *mean solar radiation* (wm^−2^), *mean wind speed* (m/s), *mean transformed wind direction* (°) as linear functions. Final models were obtained using a backwards-stepwise-selection-based procedure [39], such that variables that did not contribute significantly to explaining variation in trap catch were successively eliminated on the basis of AIC [40]. This continued until the removal of a variable caused an increase in AIC of two or more. Differences in trap catch size between covering treatments were then assessed using multiple Tukey’s all-pair comparisons using the ‘glht’ function in package multcomp version 1.3-7 [41].

In addition, for each treatment in field trial one and two the mean *Coefficient of Protection from Intrusion* (*CPI*) [46] was calculated with *CPI* = ((*A* − *B*) × 100)/*A* where *A* is the number of *Culicoides* collected inside the frame/stable with no mesh i.e. the control, and *B* is the number trapped inside the untreated mesh or the insecticide-treated mesh covered frame/stable.

## Results

### WHO cone bioassays

**Fig. 6:**
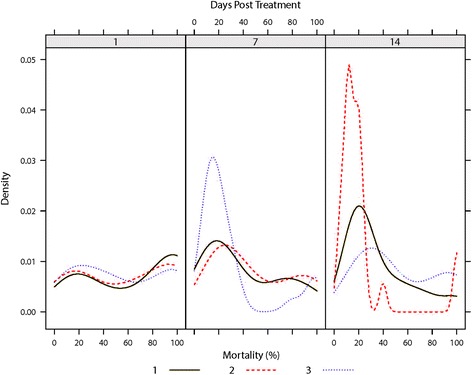
World Health Organisation (WHO) Cone Bioassays: Kernel density estimation plots illustrating the variability in *C. nubeculosus* mortality rates across treatments and mesh section replicates, split by days since treatment and treatment batches (solid green line = treatment batch 1; red dashed line = treatment batch 2; dotted blue line = treatment batch 3)

Mortality observed in exposed *C. nubeculosus* was found to be best described by a model including the predictors *treatment* and *days post-treatment* as fixed effects and *treatment batch* and *within-mesh replicate* as random factors (Table 3). Over the two week period (sampling at day 1, 7 and 14) there was no significant difference in the mortality rate observed between the untreated mesh sections (treatment K) and the filter paper controls (treatment L) (Tables 3 and 4). Mean mortality rates of 12.2 % (range: 4–16 %) and 11.1 % (range: 0–16 %) were observed in the untreated control mesh and the filter paper control, respectively. Over the two week period (sampling at day 1, 7 and 14) all insecticide/repellent-based treatments (treatments A, B, C, D, E, F, G, H, I and J) caused significantly greater mortality than either the untreated control mesh and the filter paper control (*P* ≤ 0.001) (Table 4). Treatment F, however, was the only treatment to exhibit 100 % mortality at all three sampling time points and for all treatment batches and within-mesh section replicates (Fig. 5) and caused significantly greater mortality rates than all other treatments tested (*P* ≤ 0.001) (Table 4). The mortality rates were not significantly different between the three repellent compounds tested (Treatments H, I and J) (Table 4). The mortality rates caused by exposure to the insecticide-based compounds tested (Treatments A, B, C, D, E, F and G) were significantly (*P* ≤ 0.001) higher than the repellent compounds tested (Treatments H, I, J) (Table 4). Over the two week period, mortality rates between treatment batches were relatively consistent at one day post-treatment but variation between treatment batches increased significantly at seven and 14 days post-treatment (Fig. 6). During the three minute exposure period no appreciable spatial repellence was observed in the behaviour of exposed *C. nubeculosus’* in response to any of the treatments in any of the treatment batches or within-mesh section replicates.

**Table 3:** World Health Organisation (WHO) Cone Bioassays: Regression coefficients with 95 % Wald confidence intervals and ∆AIC for the fixed effects of two final Bayesian general linear mixed models with a Binomial error distribution used to describe (*i*) mortality rate and (ii) blood feeding rate of *C. nubeculosus* (Meigen), 1830 exposed during WHO cone bioassay tests

Parameters	Mortality rate		Blood feeding rate	
	Estimate (95 % CI)	∆AIC	Estimate (95 % CI)	∆AIC
Intercept	−1.35 (−1.60; −1.11)***		−0.98 (−1.63; −0.34)**	
Treatment		1779.5		2243.4
Product B	0.72 (0.45;1.00)***		0.51 (0.02;1.00)*	
Product C	1.13 (0.85; 1.41)***		1.18 (0.72; 1.64)***	
Product D	0.53 (0.26; 0.81)***		1.12 (0.64; 1.60)	
Product E	0.17 (−0.11; 0.45)		0.93 (0.42; 1.43)***	
Product F	−5.67 (−7.74; −3.59)***		−1.99 (−5.49; 1.51)	
Product G	−0.54 (−0.84; −0.24)***		1.55 (1.02; 2.08)***	
Product H	1.89 (1.59; 2.19)***		1.69 (1.24; 2.14)***	
Product I	1.88 (1.58; 2.19)***		1.48 (1.03; 1.93)***	
Product J	1.64 (1.35; 1.93)***		0.98 (0.53; 1.44)***	
Product K	2.72 (2.38; 3.07)***		2.00 (1.55; 2.46)***	
Product L	2.83 (2.47; 3.19)***		1.77 (1.32; 2.22)***	
Days post-treatment	0.09 (0.08; 0.11)***	225.6	0.22 (0.01; 0.04)**	689.5
Mortality rate	-	-	−0.05 (−0.05; −0.04)***	463.8

Random effects included in the final model included the effect of *treatment batch* and *within section replicate*

*** *P* ≤ 0.001, ** *P* ≤ 0.01, * *P* ≤ 0.05

**Table 4:** World Health Organisation (WHO) Cone Bioassays: Multiple Tukey’s all-pair comparisons of mortality rate between treatments taking into account variation caused by days since treatment, batch and replicate

Treatment	A	B	C	D	E	F	G	H	I	J	K	L
A	-	0.72***	1.13***	0.53**	0.17 ^NS^	−5.67***	−0.54*	1.89***	1.88***	1.64***	2.72***	2.83***
B		-	0.41 ^NS^	−0.19 ^NS^	−0.56**	−6.39***	−1.26***	1.17***	1.16***	0.92***	2.00***	2.11***
C			-	−0.60***	−0.97***	−6.80***	−1.67***	0.76***	0.75***	0.50*	1.59***	1.70***
D				-	−0.37 ^NS^	−6.20***	−1.07***	1.36***	1.35***	1.10***	2.19***	2.30***
E					-	−5.83***	−0.71***	1.72***	1.71***	1.47***	2.56***	2.66***
F						-	5.13***	7.56***	7.55***	7.30***	8.39***	8.50***
G							-	2.43***	2.42***	2.18***	3.26***	3.37***
H								-	−0.01 ^NS^	−0.25 ^NS^	0.84***	0.94***
I									-	−0.24 ^NS^	0.85***	0.95***
J										-	1.09***	1.19***
K											-	0.11 ^NS^
L												-

Estimate with *P* values shown as superscript (***P ≤ 0.001, **P ≤ 0.01, *P ≤ 0.05)

**Fig. 7:**
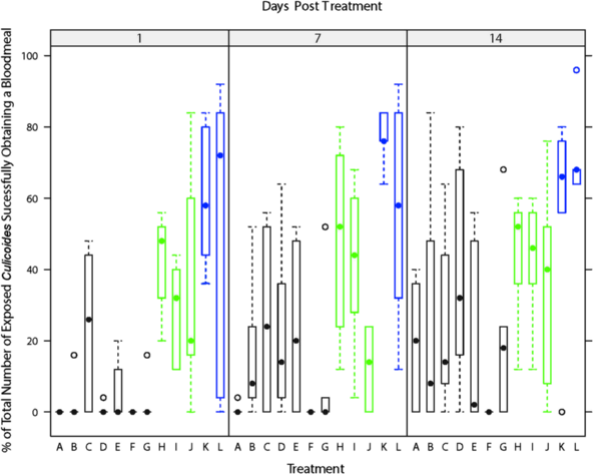
World Health Organisation (WHO) Cone Bioassays: Box-and-whisker plots of percentage of the total number of exposed *C. nubeculosus* which successfully obtained a bloodmeal split by treatment and days post-treatment. Variation within the box-and-whisker plots represents variance in blood feeding rate between treatment batches and between within-mesh section replicates. Median values shown as filled black circles, outliers as hollow black circles, the interquartile range as hollow rectangles (black: insecticide-based treatments; green: repellent-based treatments; blue: negative controls)

**Fig. 8:**
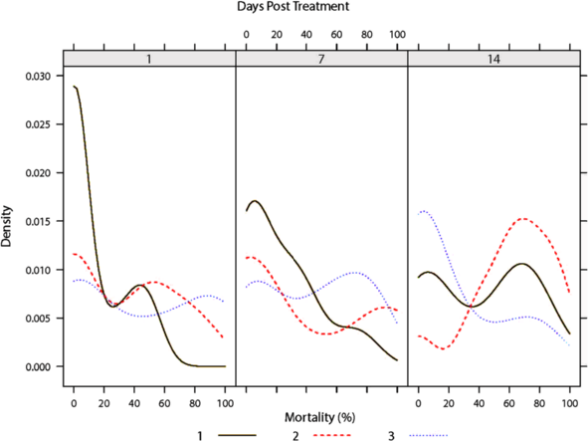
World Health Organisation (WHO) Cone Bioassays: Kernel density estimation plots illustrating the variability in *C. nubeculosus* bloodfeeding rates across treatments and mesh section replicates, split by days since treatment and treatment batches (solid green line = treatment batch 1; red dashed line = treatment batch 2; dotted blue line = treatment batch 3)

Blood feeding observed in exposed *C. nubeculosus* was best described by a model including the predictors’ *treatment, days post-treatment* and *mortality rate* as fixed effects and *treatment batch* and *within-mesh replicate* as random factors (Table 3). Over the two week period (sampling at day 1, 7 and 14) there was no significant difference in the number of *C. nubeculosus* which successfully obtained a bloodmeal, taking into account the mortality rate observed between the untreated mesh sections (treatment K) and the filter paper controls (treatment L) (Table 5). Mean blood feeding rates of surviving *C. nubeculosus* of 73.9 % (0–100.0 %) and 68.4 % (0–100.0 %) were observed in the untreated control mesh and the filter paper control, respectively (Fig. 7). Over the two week period (sampling at day 1, 7 and 14) repellent treatments H and I caused no significant reduction in blood feeding rate in surviving *C. nubeculosus*, though repellent-based treatment J did cause a significant (*P* ≤ 0.001) decrease in the rate of blood feeding for surviving *C. nubeculosus* (Table 5). Insecticide-based treatments A, B, C, D and E all demonstrated significant reductions in blood feeding rates in surviving *C. nubeculosus* when compared to the untreated mesh sections (treatment K) and the filter paper controls (treatment L) (Table 5). Over the two week period, significant variability in blood feeding rates in surviving *C. nubeculosus* between treatment batches was observed at all-time points measured (Fig. 8). Due to the superior performance of Treatment F over all other treatments in causing mortality in exposed *C. nubeculosus*, all further field-based investigations used this insecticide-based treatment.

**Table 5:** World Health Organisation (WHO) Cone Bioassays: Multiple Tukey’s all-pair comparisons of bloodfeeding rate between treatments taking into account variation caused by days since treatment, batch and replicate

Treatment	A	B	C	D	E	F	G	H	I	J	K	L
A	-	0.51	1.18***	1.12***	0.93*	−1.99	1.55***	1.69***	1.48***	0.98**	2.00***	1.77***
B		-	0.67*	0.61†	0.42	−2.50	1.04***	1.18***	0.97***	0.47	1.49***	1.26***
C			-	−0.06	−0.26	−3.17	0.37	0.51***	0.30	−0.20	0.82***	0.59*
D				-	−0.20	−3.11	0.42	0.56*	0.36	−0.14	0.88***	0.65*
E					-	−2.91	0.62	0.76**	0.55	0.06	1.08***	0.84**
F						-	3.53	3.68	3.47	2.97	3.99	3.76
G							-	0.14	−0.07	−0.56	0.46	0.22
H								-	−0.21	−0.71***	0.31	0.08
I									-	−0.50*	0.52*	0.29
J										-	1.02***	0.79***
K											-	−0.23
L												-

Estimate with *P* values shown as superscript (***P ≤ 0.001, **P ≤ 0.01, *P ≤ 0.05)

### Field trials

#### Field trial one

Over the 24 nights of UV CDC light-suction trap collections in field trial one 161 *Culicoides* comprising of five species: *C. obsoletus*, *C. scoticus*, *C. dewulfi*, *C. chiopterus* and *C. brunnicans* (Edwards), 1939 were collected (Table 6). Collections were dominated by female *Culicoides* (97 %), with only five male *C. obsoletus* collected. Of the 145 female subgenus *Avaritia Culicoides* collected 52 % were identified via multiplex PCR as *C. obsoletus*; 19 % as *C. scoticus*; 15 % as *C. dewulfi*; and 8 % as *C. chiopterus*. The multiplex PCR however, failed to identify 6 % of morphologically identified subgenus *Avaritia* specimens. Of the total number of *Culicoides* collected 94 % were collected inside the frames with no mesh covering i.e. the control, 6 % were collected inside the frames with untreated mesh coverings and no *Culicoides* were collected within the frames with insecticide-treated mesh coverings (Table 6).

**Table 6:** Field Trial One: *Culicoides* collected within Ultraviolet (UV) Center for Disease Control (CDC) light-suction traps which are within frames which are either (*i*) covered with insecticide-treated mesh, (*ii*) covered with untreated mesh, or (*iii*) left uncovered, i.e. no mesh, total number collected with the number of female; male shown in parenthesis

Treatment		*Culicoides* species				
	*C. obsoletus* (Meigen), 1818	*C. scoticus* Downes and Kettle, 1952	*C. dewulfi* (Goetghebuer), 1936	*C. chiopterus* (Meigen), 1830	Obsoletus Group	*C. brunnicans* (Edwards), 1939
Insecticide-Treated mesh	0 (0; 0)	0 (0; 0)	0 (0; 0)	0 (0; 0)	0 (0; 0)	0 (0; 0)
Untreated mesh	5 (4; 1)	1 (1; 0)	2 (2; 0)	0 (0; 0)	1 (1; 0)	1 (1; 0)
No mesh control	78 (71; 7)	27 (27; 0)	20 (20; 0)	12 (12; 0)	7 (7; 0)	7 (7; 0)

he total number of *Culicoides* collected and the total number of potential AHSV vector *Culicoides* collected by the UV CDC light-suction traps inside the wooden frames was found to be best described by a model including the predictors’ *treatment, precipitation*, *windspeed*, *transformed wind direction*, *solar radiation* and *days post-treatment* as fixed effects and *treatment batch* as a random factor (Table 7). Initial models and multiple Tukey’s all-pair comparisons indicated the UV CDC traps inside frames which were covered with either the insecticide-treated mesh (treated using treatment F see Table 1) or the untreated mesh collected significantly (*P* ≤ 0.001) fewer *Culicoides* and significantly (*P* ≤ 0.001) fewer female potential AHSV vector *Culicoides* than that collected in the uncovered frames (Table 8).However, the number of *Culicoides* and the number of female potential AHSV vector *Culicoides* collected inside frames which were covered with insecticide-treated mesh compared to the untreated mesh were not significantly different (Table 7).

**Table 7:** Field trial one: Regression coefficients with 95 % Wald confidence intervals and ∆AIC for the fixed effects of two final Bayesian general linear mixed models with a Poisson error distribution used to describe (*i*) the total number *Culicoides* collected (all species) and (*ii*) the number of females of potential AHSV vector species of *Culicoides* (*Culicoides obsoletus* (Meigen), 1818; *Culicoides scoticus* Downes and Kettle*,* 1952; *Culicoides dewulfi* (Goetghebuer), 1936; and *Culicoides chiopterus* (Meigen), 1830) collected in Ultraviolet (UV) Center for Disease Control (CDC) light-suction traps within frames either (*i*) covered with insecticide-treated mesh, (*ii*) covered with untreated mesh, or (*iii*) left uncovered

Parameters	Total *Culicoides* collected		Total female potential AHSV vectors *Culicoides*	
	Estimate (95 % CI)	∆AIC	Estimate (95 % CI)	∆AIC
Intercept	2.322 (1.79; 2.86)***		2.32 (1.79; 2.86)***	
Treatment		266..8		266.8
Insecticide-treated mesh	−5.19 (−7.37; −3.01)***		−5.19 (−7.37; −3.01)***	
Untreated mesh	−2.65 (−3.28; 2.02)***		−2.65 (−3.28; −2.02)***	
Precipitation	−3.30 (−6.11: −0.49)*	5.6	−3.30 (−6.11; −0.50)*	5.6
Windspeed	−1.35 (−2.38; −0.32)*	4.1	−1.35 (−2.38; −0.32)*	4.1
Transformed wind direction	−0.10 (−0.12: −0.32)***	116.7	−0.10 (−0.12; −0.08)***	116.7
Solar radiation	0.02 (0.00, 0.03)**	3.4	0.02 (0.00; 0.03)**	3.4
Days post-treatment	−0.11 (−0.015; −0.06)***	16.7	−0.11 (−0.15; −0.06)***	16.7

Random effects included in the final model included the effect of treatment batch

*** *P* ≤ 0.001, ** *P* ≤ 0.01, * *P* ≤ 0.05

**Table 8:** Field trial one: Multiple Tukey’s all-pair comparisons of (*i*) the total number *Culicoides* collected (all species) and (*ii*) the number of females of potential AHSV vector species of *Culicoides* (*Culicoides obsoletus* (Meigen), 1818; *Culicoides scoticus* Downes and Kettle*,* 1952; *Culicoides dewulfi* (Goetghebuer), 1936; and *Culicoides chiopterus* (Meigen), 1830) collected within Ultraviolet (UV) Center for Disease Control (CDC) light-suction traps within frames which are either (*i*) covered with insecticide-treated mesh, (*ii*) covered with untreated mesh, or (*iii*) left uncovered

		Insecticide-treated mesh	Untreated mesh	No mesh
Total *Culicoides* (all species)	Insecticide-Treated Mesh	-	−2.54 ^NS^	−5.19***
	Untreated Mesh	-	-	−2.65***
	No Mesh	-	-	-
Total female potential AHSV vector *Culicoides*	Insecticide-Treated Mesh	-	−2.54 ^NS^	−2.65***
	Untreated Mesh	-	-	−2.65***
	No Mesh	-	-	-

*NS* not significant

Estimate with *P* values shown as superscript *** P ≤ 0.001

The mean *CPI* for the untreated mesh and the insecticide-treated mesh in comparison to the no mesh control was 88 % (range: −100 %; 100 %) and 100 % (range: 100 %; 100 %) respectively.

#### Field trial two

Over the nine nights of UV CDC light-suction trap collections in field trial two, 74 *Culicoides* comprising of five species: *C. obsoletus*, *C. scoticus*, *C. dewulfi*, *C. chiopterus* and *C. brunnicans* were collected (Table 9). Collections were again dominated by female *Culicoides* (99 %), with only one male *C. brunnicans* collected. Of the 73 female subgenus *Avaritia Culicoides* collected 34 % were identified via multiplex PCR as *C. obsoletus*, 47 % as *C. scoticus* 6 % as *C. dewulfi* and 7 % as *C. chiopterus* (Table 9). The multiplex PCR however, failed to identify 7 % of morphologically identified subgenus *Avaritia* specimens. Of the total *Culicoides*, 80 % were collected inside the stables with no mesh protection, 16 % were collected inside the stables with untreated mesh protection and 4 % were collected within the stables with insecticide-treated mesh protection (Table 9).

**Table 9:** Field Trial two: *Culicoides* collected within Ultraviolet (UV) Center for Disease Control (CDC) light-suction traps within stables whose entrances were either (*i*) covered with insecticide-treated mesh, (*ii*) covered with untreated mesh, or (*iii*) left uncovered

Treatment		*Culicoides* species				
	*C. obsoletus* (Meigen), 1818	*C. scoticus* Downes and Kettle, 1952	*C. dewulfi* (Goetghebuer), 1936	*C. chiopterus* (Meigen), 1830	Obsoletus Group	*C. brunnicans* (Edwards), 1939
Insecticide-Treated mesh	0 (0; 0)	1 (1; 0)	0 (0; 0)	1 (1; 0)	1 (1; 0)	0 (0; 0)
Untreated mesh	5 (5; 0)	5 (5; 0)	0 (0; 0)	1 (1; 0)	1 (1; 0)	0 (0; 0)
No mesh control	20 (20; 0)	28 (28; 0)	4 (4; 0)	3 (3; 0)	3 (3; 0)	1 (1; 0)

Total number collected with the number of female; male shown in parenthesis

Both the total number of *Culicoides* collected and the total number of subgenus *Avaritia* individuals collected within the stables was found to be best described by a model including *treatment* and *windspeed* as fixed effects and *treatment batch* as a random factor (Table 10). Initial models and multiple Tukey’s all-pair comparisons indicated the UV CDC trap inside stables whose entrances were covered with either the insecticide-treated mesh (treated using treatment F see Table 1) or the untreated mesh collected significantly (*P* ≤ 0.001) fewer *Culicoides* and significantly (*P* ≤ 0.001) fewer subgenus *Avaritia* individuals than that collected in the uncovered control stable (Table 11). However, the number of *Culicoides* and the number of subgenus *Avaritia* collected inside stables whose entrances were covered with insecticide-treated mesh in comparison to the untreated mesh were not significantly different (Table 7).

**Table 10:** Field trial two: Regression coefficients with 95 % Wald confidence intervals and ∆AIC for the fixed effects of two final Bayesian general linear mixed models with a Poisson error distribution. The model was used to describe (*i*) the total number *Culicoides* collected (all species) and (*ii*) the number of females of potential AHSV vector species of *Culicoides* (*Culicoides obsoletus* (Meigen), 1818; *Culicoides scoticus* Downes and Kettle*,* 1952; *Culicoides dewulfi* (Goetghebuer), 1936; and *Culicoides chiopterus* (Meigen), 1830) collected within Ultraviolet (UV) Center for Disease Control (CDC) light-suction traps within stables whose entrances are either (*i*) covered with insecticide-treated mesh, (*ii*) covered with untreated mesh, or (*iii*) left uncovered

Parameters	Total *Culicoides* collected		Total female potential AHSV vector *Culicoides*	
	Estimate (95 % CI)	∆AIC	Estimate (95 % CI)	∆AIC
Intercept	4.13 (2.91; 5.35)***		4.27 (2.91; 5.35)***	
Treatment		68.7		68.9
Insecticide-treated mesh	−2.83 (−3.91; −1.74)***		−2.81 (−3.91; −1.74)***	
Untreated mesh	−1.56 (−2.18; −0.94)***		−1.54 (−2.18; −0.94)***	
Windspeed	−0.06 (−0.09; −0.04)***	62.9	−0.07 (−0.09; −0.04)***	63.08

Random effects included in the final model included the effect of treatment batch

*** *P* ≤ 0.001

**Table 11:** Field trial two: Multiple Tukey’s all-pair comparisons of (*i*) the total number *Culicoides* collected (all species) and (*ii*) the total number of female potential AHSV vector species of *Culicoides* (*Culicoides obsoletus* (Meigen), 1818; *Culicoides scoticus* Downes and Kettle*,* 1952; *Culicoides dewulfi* (Goetghebuer), 1936; and *Culicoides chiopterus* (Meigen), 1830) collected within Ultraviolet (UV) Center for Disease Control (CDC) light-suction traps which are within stables whose entrances are either (*i*) covered with insecticide-treated mesh, (*ii*) covered with untreated mesh, or (*iii*) left uncovered, i.e. no mesh

		Insecticide-treated mesh	Untreated mesh	No mesh control
Total *Culicoides*	Insecticide-Treated Mesh	-	1.27^NS^	−2.83***
	Untreated Mesh	-	-	−1.56***
	No Mesh Control	-	-	-
Total female potential AHSV vector *Culicoides*	Insecticide-Treated Mesh	-	1.27^NS^	−2.83***
	Untreated Mesh	-	-	−1.56***
	No Mesh Control	-	-	-

*** *P* ≤ 0.001

**Table 12:** Meteorological conditions recorded during field trial one and two. Mean with range shown in parenthesis

Variable	Field trial one	Field trial two
Air temperature (°C)	24.9 (10.2–19.6)	17.5 (14.4–20.3)
Relative humidity (%)	87.7 (73.1–99.5)	81.9 (75.0–89.0)
Rainfall (mm)	0.0 (0.0–0.3)	0.0 (0.0–0.2)
Wind speed (ms^−1^)	0.4 (0.2–1.3)	0.5 (0.3–0.9)
Solar radiation (wm^-2^)	11.2 (3.4–93.2)	54.0 (24.4–75.4)

The mean *CPI* for the untreated mesh and the insecticide-treated mesh in comparison to the no mesh control was 71 % (range: 0 %; 100 %) and 96 % (range: 78 %; 96 %) respectively. Meteorological conditions recorded during field trial one and two are shown in Table 12.

## Discussion

This study is the first to utilise WHO cone bioassays to investigate the mortality rate in *Culicoides* caused by exposure to insecticide treated nets (ITNs). In addition, the study is also the first to investigate the effectiveness and logistical feasibility of utilising ITNs to protect horses from *Culicoides* in the UK using field experiments. A pyrethroid-based insecticide which is currently licenced for use by amateurs and commercially available ready-formulated on the UK market (treatment F: Tri-Tec 14® (LS Sales (Farnham) Ltd, UK)) was found to cause 100 % mortality in exposed *Culicoides* for up to two weeks post-treatment in the WHO cone bioassays. Subsequently, untreated-mesh and mesh treated with the insecticide Tri-Tec 14® were found to significantly reduce the entry of *Culicoides* both into frames covered with mesh and to stables whose entrance had been covered with mesh. These results provide strong quantitative evidence that this relatively straightforward measure can have a significant impact on reducing *Culicoides*-horse contact and therefore at least provide a substantial degree of mitigation against AHSV transmission.

In addition to testing insecticidal compounds, this study also examined the impact of repellents on *C. nubeculosus* survival following exposure. Repellent-based compounds are commonly used by horse-owners in attempts to reduce biting rates on horses, both during grazing and while being ridden. It is therefore likely that at least a proportion of owners might treat mesh netting with these same products to reduce the impact of equine summer seasonal recurrent allergic dermatitis or in the event of an outbreak of AHSV. While, as expected, the three repellent-based products tested were not found to cause any significant levels of mortality in *C. nubeculosus* following exposure, the Citradiol-based repellent (Treatment J: NAF Off Extra Effect (Greencoat Ltd, UK)) did appear to result in a significant anti-feeding response in exposed *Culicoides* at 1, 7 and 14 days post-treatment of the mesh netting, which is worthy of further investigation.

The use of untreated mesh to screen stables has previously been shown to provide a degree of protection to equines from *Culicoides* [20, 47, 48]. The use of synthetic pyrethroids to further enhance the protection provided by screening for equines was supported by this study reinforcing the findings of Pages et al. [49, 50]. However, the results of this study highlight the significant variation in performance among those formulations licensed for amateur use. All insecticide-based treatments tested within this study contained either cypermethrin, pyrethrins, permethrin, tetramethrin or a combination of these compounds. While within this study there was no clear order of effectiveness in causing mortality and/or anti-feedant effect between the treatments according to what pyrethroid type they contained, systematic comparison of active ingredients would assist product development. An obvious omission to the above list is any insecticide based on deltamethrin, which has demonstrated high toxicity to *C. obsoletus* in Spain and France in laboratory exposure assays to treated filter papers [51, 52], but is not currently available in the UK in a formulation available for amateur use. Robin et al. [53], however, found ‘off-label’ topical application of 1 % deltamethrin did not significantly reducing biting rates on horses. The treatment identified as causing the highest mortality in the WHO cone bioassay tests (treatment F: Tri-Tec 14® (LS Sales (Farnham) Ltd, UK)) did have the highest concentration of cypermethrin (0.15 % *w/w*) in comparison to the other cypermethrin-based formulations tested, this is however a significantly lower concentration than the 5.93 % *w/w* alphacypermethin treatment tested by Pages et al. [49, 50]. Further dose-dependent investigations into the sub-lethal effects of exposure to cypermethrin and other pyrethroid types on both feeding response and host-location are required, as have previously been conducted to investigate dose-dependent mortality rates in *Culicoides* [51, 54].

The manufacturers recommended re-spray period for Tri-Tec 14® is 14 days and environmentally exposed mesh netting was found to still be 100 % effective in killing *C. nubeculosus* at this time. A respray interval of two weeks is likely to be both logistically feasible and financially viable; however, the ability for stables entrances to be covered effectively is likely to vary widely due to variations in stable design between and within yards. The apparent reduction in the level of protection provided in field trial one compared to field trial two by the untreated mesh (*CPI*:87.9 and 70.95 % respectively) and the insecticide-treated mesh (*CPI*: 100 and 96.3 %) are likely due to the increased logistical difficulties in covering all entrance gaps to the stable consistently and the small proportion of *Culicoides* exhibiting diurnal activity. Nonetheless, the reduction of *Culicoides* collected within the insecticide-treated net screened stable to a single individual in the second field trial is similar to results in RSA [20] and illustrates the value of this technique in mitigation.

Porter [55] found that while *Culicoides* can pass through untreated insect screens with mesh sizes of 1.6 mm^2^ they did reduce *Culicoides* entry rates by 56 %. The use of smaller aperture mesh to that used in this study may have an increased ability to exclude *Culicoides* from an area by acting as a physical barrier i.e. the mesh aperture is too small for the *Culicoides* to pass through. The use of such fine mesh is, however, also associated with a significant reduction in airflow through stables which has previously been found to have a detrimental effect on the welfare and respiratory performance of horses (reviewed by [56]), a particular concern regarding high-performance competition horses. The selection of a net with a moderately large aperture in this study allows the detrimental effects on airflow to be minimised while still reducing *Culicoides* entry rates. The contact insecticidal effect of using treated netting provides a second line of defence with the quick and effective knockdown provided by the Tri-Tec 14® reducing the risk of biting and therefore transmission by *Culicoides* that are able to pass through the netting.

Braverman et al. [57] are the only study to have previously investigated the efficacy of netting treated with Tri-Tec 14®. In contrast to the findings of this study Tri-Tec 14® treated netting was found to only significantly reduce collections in Israel of *C. imicola* for between two to five hours post-treatment (applied at 0.008 g/m^2^) in comparison to untreated net. This earlier study, however, utilized an uncoated polyester type net (aperture 1.3 mm), in comparison to the polyvinyl-coated polyester mesh (1.6 mm aperture) used in the present study. Further investigation is required to evaluate the level and duration of effect of the Tri-Tec 14® treatment between different net materials, for different vector species and in different climates. A key consideration is the degree to which net aperture impacts upon air circulation and dust collection on the net which has the potential to inhibit the insecticide’s performance [18]. In addition, while there has been limited investigation into the presence of insecticidal resistance in *Culicoides* this phenomenon is well documented in other vector groups and further investigation is required into the mechanisms underlying the development of resistance in *Culicoides* and how this varies both between species and at a population level.

ZeroFly® (Vestergaard Frandsen, Lausanne, Switzerland) is an insect screen material available pre-treated with deltamethrin, and is used for large biting fly control in Africa and Asia. While it is not currently commercial available in Europe preliminary, trials in Europe with *Culicoides* indicate that this type of net may be a potential alternative to stakeholders treating nets themselves. Further studies to compare ZeroFly®’s effectiveness and duration of activity with the Tri-Tec 14® treated mesh net combination tested in this study, would significantly inform outbreak response strategies.

While species of *Culicoides* are considered to be the principal vector of AHSV [7], large-scale infection studies using horses did demonstrate that transmission by other vectors including mosquitoes is possible however, this is likely to be a rare event (reviewed by [58]). Further research is needed to examine the vector competence and more widely the vector capacity of Palaearctic *Culicoides* species for AHSV in addition to the potential role of other Palaearctic hematophagous arthropods in AHSV transmission. If other hematophagous arthropods are found to be epidemiologically significant vectors of AHSV their activity and response to vector control measures e.g. insecticide resistance must also be a considered as part of an integrative vector control program in the event of an AHSV outbreak.

The infection of a susceptible uninfected host when fed on by an infected *Culicoides* is a highly efficient process, however, multiple barriers to infection are present in the opposing process of the infection of an uninfected vector which feeds on an infected host (reviewed by [59]). Therefore, while the prevention of the infection of an individual animal may require the complete prevention of *Culicoides* biting to prevent infection, a vector control measure which is significantly less effective at preventing biting my still have epidemiologically significant effect on the rate of transmission of AHSV. While logistically difficult to quantify in a field situation efforts to estimate the community level and cumulative effects of different vector control measures on the outcome of AHSV outbreaks e.g. the rate of spread, are an important consideration when planning outbreak response policies.

## Conclusions

This study has shown that ITNs have the potential to offer protection to horses from *Culicoides* in the event of an AHSV epidemic, during international movement through endemic regions for competition and racing, and in reducing biting nuisance. This study has, however, demonstrated that there is significant variation in the effectiveness of different commercially available insecticide-based treatments when used to treat mesh and that insecticides providing 100 % mortality in WHO cone bioassay tests do not necessarily provide complete prevention of ingression in a field environment. Vector-protected accommodation created using ITNs can be utilised as part of an integrated control program, perhaps in combination with regular applications of topical repellents and insecticide treatment within stables. While the numbers of *Culicoides* collected in this study is consistent with that collected at other equine sites in the UK (Harrup, unpublished data), further investigation of the effectiveness of ITNs for preventing ingress of *Culicoides* to equine accommodation at high abundance field sites e.g. those where horses and cattle are co-located, would be very insightful; in addition, to investigations into the effectiveness of commercially available topical repellents and/or insecticides in reducing equine-vector contact rates. While challenging to perform, studies investigating if any reduction in vector-host contact caused by ITN usage in AHSV hyper-endemic regions could significantly reduce infection rates would be invaluable when considering the limitations of such control measures [1, 60].
